# Comparing Neuromorphic Solutions in Action: Implementing a Bio-Inspired Solution to a Benchmark Classification Task on Three Parallel-Computing Platforms

**DOI:** 10.3389/fnins.2015.00491

**Published:** 2016-01-08

**Authors:** Alan Diamond, Thomas Nowotny, Michael Schmuker

**Affiliations:** School of Engineering and Informatics, University of SussexBrighton, UK

**Keywords:** neuromorphic hardware, benchmarking, bioinspired, spiking neural networks, classification

## Abstract

Neuromorphic computing employs models of neuronal circuits to solve computing problems. Neuromorphic hardware systems are now becoming more widely available and “neuromorphic algorithms” are being developed. As they are maturing toward deployment in general research environments, it becomes important to assess and compare them in the context of the applications they are meant to solve. This should encompass not just task performance, but also ease of implementation, speed of processing, scalability, and power efficiency. Here, we report our practical experience of implementing a bio-inspired, spiking network for multivariate classification on three different platforms: the hybrid digital/analog Spikey system, the digital spike-based SpiNNaker system, and GeNN, a meta-compiler for parallel GPU hardware. We assess performance using a standard hand-written digit classification task. We found that whilst a different implementation approach was required for each platform, classification performances remained in line. This suggests that all three implementations were able to exercise the model's ability to solve the task rather than exposing inherent platform limits, although differences emerged when capacity was approached. With respect to execution speed and power consumption, we found that for each platform a large fraction of the computing time was spent outside of the neuromorphic device, on the host machine. Time was spent in a range of combinations of preparing the model, encoding suitable input spiking data, shifting data, and decoding spike-encoded results. This is also where a large proportion of the total power was consumed, most markedly for the SpiNNaker and Spikey systems. We conclude that the simulation efficiency advantage of the assessed specialized hardware systems is easily lost in excessive host-device communication, or non-neuronal parts of the computation. These results emphasize the need to optimize the host-device communication architecture for scalability, maximum throughput, and minimum latency. Moreover, our results indicate that special attention should be paid to minimize host-device communication when designing and implementing networks for efficient neuromorphic computing.

## Introduction

New massively parallel neuromorphic systems may have the potential to deliver engineering solutions to general computing problems by leveraging, often bio-inspired, neuronal models (Boahen, 2006; Indiveri et al., 2011). The ability of animals to solve complex perceptual and behavioral tasks with low power requirements suggests that, via a suitable platform, this approach might ultimately prove superior, for some tasks, to classical AI on conventional processors in terms of effective performance, speed or power consumption. However, executing a spiking neuronal simulation with even a fraction of the biological scale and synaptic connectivity on a conventional computing platform requires considerable computing resources. This is often further compounded by demanding additional requirements such as real time execution or manageable power consumption and heat generation (Hasler and Marr, 2013).

To meet the challenge of efficient neuromorphic computing a number of neuromorphic platforms have been—and continue to be—developed. Some of these systems employ analog neuron circuits (see Indiveri et al., 2011, for a review), others are implemented in FPGAs (e.g., Pearce et al., 2013), or employ specialized digital hardware, such as the SpiNNaker system (Khan et al., 2008; Furber et al., 2013). Moreover, GPU-based simulators have recently become popular for neuromorphic computing because they can provide a considerable speedup over CPU-based simulators on desktop systems, whilst retaining a manageable power budget and are fully programmable using high-level languages (Fidjeland and Shanahan, 2010; Nowotny et al., 2014). Successful use cases for neuromorphic computing have been reported for diverse applications like image recognition, for example on IBM's TrueNorth platform (Merolla et al., 2014) and on SpiNNaker (Serrano-Gotarredona et al., 2015), but also for modeling a variety of brain circuits (Pfeil et al., 2013), and generic multivariate classification (Schmuker et al., 2014).

Amid this widening landscape, researchers seeking to investigate the behavior of their, often large-scale, spiking models on a given task face the problem of choosing the approach that is best suited to their use-case. Logically, it follows that an informed approach to platform selection would consider the suitability of alternatives in the context of the task in question. However, it is clear that a published specification or feature sheet cannot fully capture all the pertinent information that is required. Rather, a hands-on evaluation of the candidate system is required to make a fully informed choice.

Tools for an unbiased comparison of neuromorphic approaches are available, most notably the PyNN meta-language that has been developed as a part of the FACETS project (Davison et al., 2008). Its goal is to provide a common programming interface that allows running a single network design on several platforms. In theory, PyNN thus enables a truly unbiased comparison of neuromorphic backends when a benchmark network is used that is equally well supported on all considered platforms. However, each platform has unique features that may be useful in providing a tailored solution to the computing problem at hand. Since a network that runs without modification on all platforms is naturally restricted to the smallest common feature set, it may be quite limited and fail to reveal the essential advantages of the various platforms (and potential disadvantages) for the use case at hand.

Here, we use an alternative approach to assessing neuromorphic platforms: Instead of defining a network that runs equally well on all compared platforms, we ported the *concept* of a functional spiking network model for generic pattern recognition to each platform, with the aim to exploit each platform's individual advantages. The classifier network was based on a spiking model of the insect olfactory system (Schmuker and Schneider, 2007; Schmuker et al., 2014). We tested three platforms: the mixed-signal, accelerated Spikey system (Pfeil et al., 2013), the digital SpiNNaker system (Khan et al., 2008; Furber et al., 2013), and the GPU-based GeNN simulator (Nowotny, 2011; Nowotny et al., 2014). To compare the resulting performance, we applied the classifier implementation for each platform to the MNIST digit classification task (http://yann.lecun.com/exdb/mnist/). MNIST was chosen as a standard, non-trivial classification problem, which must practically accommodate both a high number of samples (tens of thousands)—thus exercising simulation speed capabilities—and high dimensionality (images are grayscale, 28 × 28 pixels = 784 dimensions), thus exercising speed, capacity and power requirements. It should be noted that the Spikey platform supports only a small neuron count (192) compared to the other platforms which, when configured for this model, support emulation of up to 12,000 (SpiNNaker SpiNN-3 board) or 18,000 (GeNN—Titan Black GPU) of neurons in real time or faster. Hence, compromises were required regarding the model scale implemented on Spikey. Nevertheless, since this system represents the family of highly accelerated neuromorphic systems, running at a 10^4^ speedup factor compared to biological real time, it provides an interesting comparison with regard to upcoming large-scale accelerated systems (Schemmel et al., 2010). Moreover, it is one of the designated “dissemination systems” (together with SpiNNaker) provided by the EU Flagship initiative “Human Brain Project” to external users who want to experiment with neuromorphic hardware.

We report on the practical experience of implementation, on modifications that were necessary to provide the same functionality, and on unique features of each platform that we exploited to improve the function of the network. We provide comparative metrics such as inclusive end-to-end execution speed, the profile of power drawn and the overall energy consumption. Power and energy measurements are particularly relevant in the neuromorphic domain since neuromorphic platforms are believed to be capable of delivering large scale spiking models to projects with specific requirements for scalability of performance or low power consumption, such as robotics (Khan et al., 2008; Hasler and Marr, 2013).

The remainder of the paper is presented with the following structure. In Section Methods, we summarize the conceptual model for the bio-inspired classifier, briefly review the architecture of the three employed neuromorphic platforms, and report on how we transformed the conceptual model into a functional experimental classifier, comparing the resulting implementations. In Section Results, we present comparative metrics covering classification performance, execution speed, power consumption, and total energy use. For speed and power, in recognition that model simulation time alone is, by no means, the whole story we include a benchmarked breakdown of the various stages of learning and testing using as much granularity as was available on each platform. In Section Discussion, we discuss and compare the findings for each metric. We also discuss the differing implementations and look to assign these differences to current software feature sets, current hardware capabilities, and fundamental architectural approach differences.

## Methods

### Classifier design

To compare the neuromorphic platforms we draw upon a genuine research question, namely the capabilities of a generic multivariate classifier based on a spiking neural network model abstracted from the insect olfactory system, in particular the antennal lobe (AL). This design has been previously described and investigated in detail (Schmuker and Schneider, 2007; Diamond et al., 2014; Schmuker et al., 2014), so we provide here only a summary of the conceptual design.

In insects, broadly tuned receptor responses are passed via olfactory receptor neurons (ORNs) to the AL, where they converge on spherical areas of high synaptic connectivity, the so-called “glomeruli” (Couto et al., 2005; Hallem and Carlson, 2006; Tanaka et al., 2012). Each glomerulus collects information relayed by one class of olfactory receptor and thus represents one channel of olfactory information entering the system. This information is filtered in the AL by lateral inhibition between glomeruli to generate a sparser representation with higher contrast between similar stimuli, intended to enable separation of closely related odors. The AL projects its output to higher brain areas where classification and multisensory integration take place (Heisenberg, 2003; Strutz et al., 2014).

**Figure 1:**
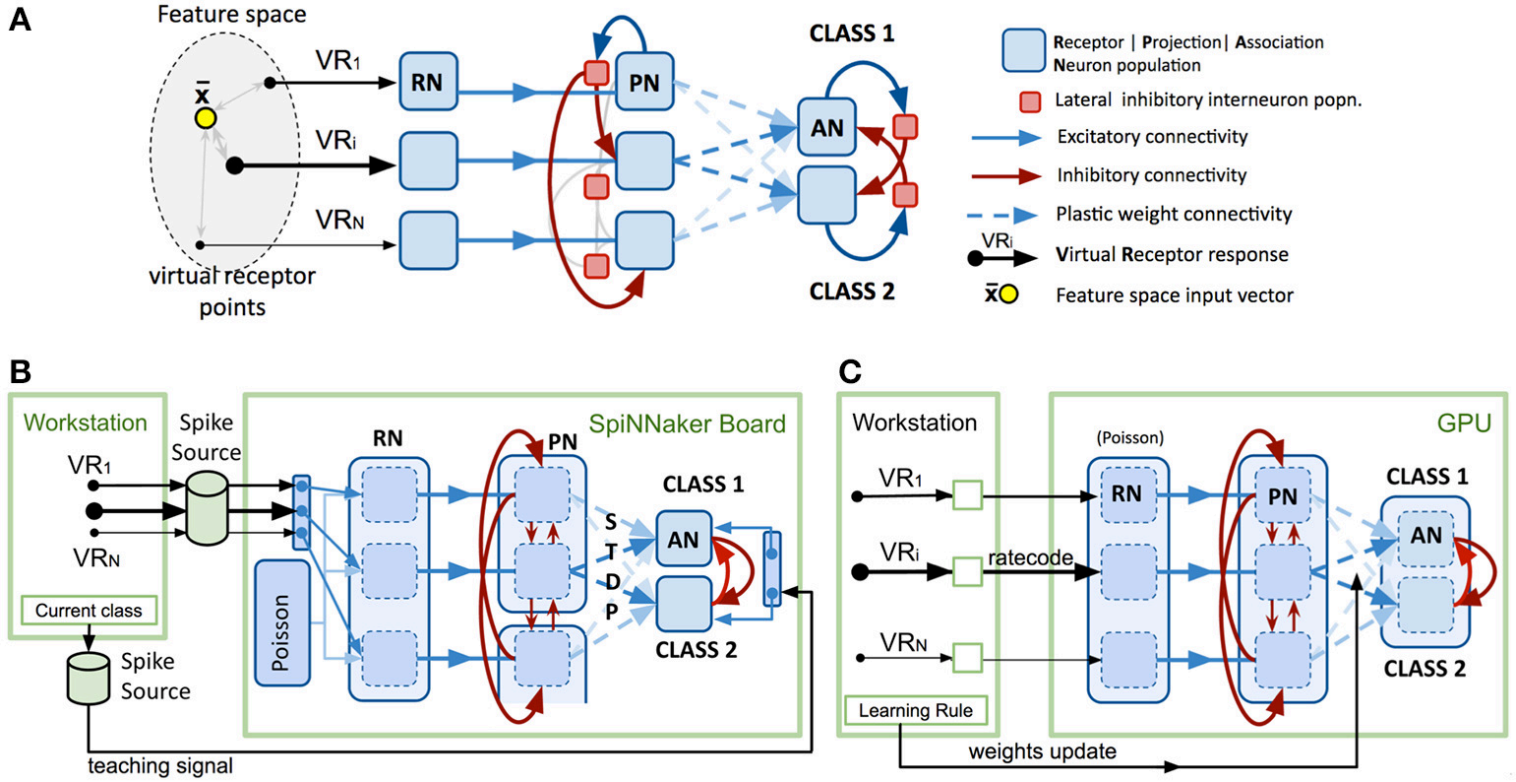
**Spiking model design and platform-specific implementations. (A)** Conceptual spiking network model for a generic multivariate classifier design based on the insect olfactory system. **(B)** Neuromorphic implementation of the model using PyNN and SpiNNaker SpiNN-3 board. See text for functional detail. Note that spike sources comprise files generated on the host workstation then transferred to the SpiNNaker board. **(C)** Neuromorphic implementation of the model using GeNN and nVidia “Titan Black” GPU card. See text for functional detail. Note that the lighter arrows are included to imply the repeated sets of connections applied to the remaining permutations of population connections.

Figure 1A shows a model intended to abstract the described bio-based strategy for any data feature space of interest. The first layer (VRs/RNs) is designed to encode multivariate, real-valued data samples into a population-based, positive, bounded, firing-rate representation. Instead of chemical sensors we employ “virtual” receptors (VRs) which each respond proportional to their proximity to the current data input, in effect encoding the data using cone-shaped radial basis functions with large, overlapping receptive fields. The centroids of the basis functions (the VR points) were placed using a self-organizing process (the current implementation uses the “neural gas” algorithm) (Martinetz and Schulten, 1995) to map the feature space described by a data set. A population of “receptor neurons” (RN) is assigned to each VR. The response of the VR to an input determines the net rate of firing for the population of RNs, which individually are described by, for example, Poisson or Gamma processes. Each population of RNs excites a matching population of “projection neurons” (PNs), which in turn send their spikes to one population of local inhibitory neurons (LNs). Each LN population sends inhibitory projections to all other PN populations in the second layer, exerting lateral inhibition, reducing correlation between VR channels and sparsening the representation of the multi-dimensional pattern.

PNs send collateral projections into the third layer of “association neuron” (AN) populations, which form a winner-take-all decision circuit through strong lateral inhibition, implemented by a second set of inhibitory local interneuron groups. Each AN population is assigned to represent one label or class in the data set (e.g., the digit “5” or the digit “7”). Highest activity in an AN population is taken to imply the presence of an example of that class at the network input.

PN activity is trained to stimulate the correct output AN population by linking them with plastic connections. Connection strengths are adjusted during exposure to a training data set via a learning rule, such as reinforcement learning or Hebbian learning.

### Neuromorphic platforms and implementations

The GeNN and Spikey implementations have been described in detail previously (Diamond et al., 2014; Schmuker et al., 2014), and are only briefly reviewed here. The SpiNNaker implementation is genuine to this publication and thus described in more detail.

### Implementation on SpiNNaker

The SpiNNaker neuromorphic platform (Khan et al., 2008; Furber et al., 2013) comprises arrays of low-power, parallel custom chips (each containing 18 ARM9 cores) running a digital software simulation of neurons and synapses. The processors are packaged on boards of 4 (SpiNN-3 board) or 48 (SpiNN-5 board) processors. The larger board is designed to be extendable by connecting further boards, racks or full cabinets. As well as low power usage, the system's philosophy focuses on the core challenge of large brain simulation to maintain spike communication in real time whilst scaling up to biological scale. To this end, a custom hardware and software packet distribution mechanism is incorporated which tolerates packet loss at higher loads for the goal of maintaining real time simulation. It is argued that this potential for loss in fact approximates the probabilistic nature of unreliable spike transmission in the brain and that effective function can nevertheless be maintained with large spike volumes (Khan et al., 2008; Furber et al., 2013). In contrast to many other simulation platforms, the spike-event focus of the architecture means that axonal delays can be employed widely and configured in detail with little cost. A standard SpiNNaker neural model is primarily configured through the provision of the “sPyNNaker” implementation (Rowley et al., 2015) of the Python-based PyNN (Davison et al., 2008) modeling framework.

For our sPyNNaker implementation (Figure 1B) the system is first configured using PyNN (version 0.7.5) in terms of neuron populations and connectivity with input spike data provided by pre-built spike-time sources combined with preset probabilistic spiking populations (see Section Data Input Formation below). The simulation is then set to run for a fixed period of time after which the record of spikes that have occurred in recorded populations can be extracted via PyNN functions and interrogated. To perform another run requires the repetition of this complete cycle, including model configuration.

To match this approach, we use just two extended runs of the model, firstly exposing it sequentially to the complete training dataset, then again to the complete test dataset. During training, the model is constructed with plastic synapses enabled between the PN and AN populations. At the end of the training run the resultant PN-AN weight matrix is extracted. The testing stage re-constructs the model with non-plastic synapses, using the fixed PN-AN weights taken from the saved matrix. The simulation is run again, this time being exposed to the entire test set. To obtain a performance score, the spike-time record of the output AN populations is then downloaded and interrogated to determine if the “winning” (highest spiking) population coincided with the correct class for each of the test data inputs.

To maximize capacity, we used the simplest standard neuron and synapse models available in sPyNNaker, the leaky integrate-fire (LIF) neuron model (Rast et al., 2010) and exponential current-based synapses. The model timestep was set to 1 ms.

To minimize the number of neuron populations and connections that needed to be simulated we abstracted the model further by replacing the inhibitory interneuron populations from the biological model with direct inhibitory synapses between PNs. We found that configuring neuron populations efficiently for SpiNNaker requires balancing a number of factors in order to not severely comprise the model size accommodated on the board. Figure 1B illustrates the configurations selected for the RN, PN, and AN layers in order to utilize the board as fully as possible.

Firstly, each core can currently only contribute to one PyNN population, thus numerous small populations (we used 30-neuron clusters per VR and per class) would rapidly use up cores whilst wasting capacity on each. In fact, the maximum number of neurons is accommodated if just a single large population is stipulated and automatically distributed across multiple cores, each handling up to 256 LIF neurons. This approach was employed for the RNs, using the connectivity matrix instead to demarcate virtual 30-neuron “clusters” representing each VR within the main RN population.

Conversely, creating a hardware-managed packet routing configuration for complex connectivity between two large populations is currently taxing for the configuration software. As the PN layer connects all-to-all with AN, we therefore ease this task by dividing the PN layer into separate PyNN populations, each comprising up to 8 × 30-neuron VR clusters.

Thirdly, neurons with afferent synapses implementing spike-timing-dependent-plasticity (STDP) require more CPU and memory resources on their parent chip, considerably reducing the number of such neurons accommodated per core (~32 currently). We therefore employed a separate PyNN population for each AN class cluster (30 neurons). This also reduces the complexity of connectivity from the PN population.

#### Data input formation

The workstation first generates the required set of N × VR points in feature space by applying the neural gas algorithm to the training data set (alone). The dataset is then traversed, obtaining a scalar, proximity-based VR response for each input in the set (see Schmuker et al., 2014 for details of the response function).

As SpiNNaker currently offers stochastic spiking populations based only on a fixed net rate, another approach was required to obtain RN populations with net spiking rates that change with the classifier input. We therefore generated, on the workstation, a spike source file which stipulated spike times for exactly N_VR_ neurons, covering the complete period of exposing the whole training or test dataset to the classifier. As we move through the dataset, the spiking rate for each of these N_VR_ neurons is set to be proportional to the response of its corresponding VR to the current sample. For each sample, the spike rate is maintained for a learning period of 120 ms—a duration determined empirically as the minimum below which classifier performance was found to degrade.

However, this rate coding only dictates the spike rate of a single neuron for each VR. In order for the spiking of these individual neurons to dictate the probabilistic net spiking rate of 30-neuron clusters we combined the spiking of each rate code neuron with that of a random selection of 30 neurons taken from a single shared 60 neuron pool of Poisson neurons firing at constant average frequency (details of this technique are provided in the Supplementary Material).

#### Plasticity and learning

On SpiNNaker, the simulation is run for the entire dataset before control returns to the workstation. This precludes the use of custom learning rules implemented on the workstation, such as those employed for both the GeNN and Spikey platforms. In principle, our host-dependent learning rule could be approximated with a “three-factor” learning rule, that is, a synaptic learning rule that takes into account pre- and postsynaptic activity plus an external reinforcement signal that governs the direction of weight plasticity (Izhikevich, 2007; Porr et al., 2007) However, sPyNNaker currently does not provide an implementation of a three-factor learning rule. We thus replaced the learning rule of the conceptual model with an implementation that achieves a similar plasticity rule using the intrinsic STDP mechanism on the SpiNNaker system paired with targeted activations of post-synaptic neurons (see also Galluppi et al., 2015). In our approach, we adjusted STDP parameters to represent a symmetric STDP curve with positive weight changes for any order of spike pairing (see Supplementary Material for more details). This implements simple Hebbian learning (Hebb, 1949), creating associations between PN (VRs) and AN (classes) that are co-active. Before training, PN-AN weights were initialized at zero weight. Then, as each input was presented, we used a second synchronized spike source to provide a teaching signal to externally stimulate concurrent activity in the correct class population of output neurons (see Figure 1B). Employing an STDP plasticity curve with positive weight changes of the same magnitude for closely paired pre and post-synaptic spikes, irrespective of the spike order, (see Supplementary Material for details) hence led to strengthening of synapses between active PNs and the neurons of the correct output class. The combination of strong WTA connectivity between AN neuron populations, a narrow window of positive weight changes in the STDP curve and the introduction of a 20 ms “silence” gap between presented inputs helped to minimize inappropriate weight changes due to accidental pairings of spikes in PNs and ANs.

### Implementation on GeNN

The GPU enhanced Neuronal Network (GeNN) meta-compiler (Nowotny, 2011; Nowotny et al., 2014) was developed to address the need for both neuronal modeling flexibility and simulation performance by drawing upon the computational power, low-cost, and wide availability offered by highly parallel general purpose graphics processing units (GP-GPU) running the Compute Unified Device Architecture (CUDA). GeNN provides a function library for a standard C/C++ environment from which neuronal model can be defined. The meta-complier is then invoked, which builds two optimized CUDA kernels, one for the neurons' state update per timestep and another for the synapses.' These act together to simulate the neuronal network in a parallel fashion, spawning a thread per neuron (neuron kernel) or per post-synaptic neuron (synapse kernel). The meta-compiler also generates a series of helper functions allowing the user to upload input data, step the simulation and extract resultant spike events or other variables (such as membrane potentials). The meta-compiler furthermore interrogates the deployment target GPU and automatically applies the manufacturer's optimization rules to the combination of its hardware properties and the model details to select appropriate CUDA deployment parameters (thread block size, shared memory allocation, etc). This eliminates one of the main constraints of effective CUDA coding—the fact that greatly suboptimal performance can easily occur if in-depth knowledge of the CUDA programming model and the properties of the deployment hardware is lacking or out of date. Neuron or synapse models can be user-specified in GeNN using an internal nomenclature. Standard examples such as Hodgkin-Huxley or Izhikevich models are available pre-rolled. Axonal delays are supported at a population-level but are expensive in memory requirements.

Our GeNN (version 2.1) implementation is illustrated in Figure 1C, and it follows essentially our previous implementation of this design (Diamond et al., 2014). The approach draws upon the ability that, after configuration and meta-compilation, the simulation can be stepped under control of the workstation whilst the complete state is retained on the device. Unlike SpiNNaker, there is no concept of real time, the entire simulation is run as fast as possible unless the user adds their own code to throttle it back.

As GeNN kernels are less efficient managing numerous separate populations, the model was configured using a single neuron population for each layer—RN, PN and AN. Thirty-neuron VR and class clusters within these are demarcated using appropriate connection matrices. The RN layer uses Poisson neurons whose net firing rate is individually controllable by an uploaded block of rate code values. The PN and AN layers use lightweight “map” neurons (Rulkov, 2002) to maximize throughput. The model timestep was set at 0.5 ms. As with SpiNNaker, for efficiency, inhibitory interneuron populations are abstracted out by using direct inhibitory synapses. Starting weights for the plastic PN-AN synapses are initialized randomly. Fixed synapse weight values and connection density between populations are detailed in Schmuker et al. (2014).

For each input in the dataset the VR's responses are calculated then converted to a set of rate codes, which are uploaded to the device to drive the RN neurons. The VR radial basis response function is detailed in Schmuker et al. (2014). The simulation is then stepped for a duration of 500 ms (simulated), being the minimum exposure time for the plasticity learning rule to remain effective. At every timestep, the spike events occurring in PN and AN layers are extracted from the device as the ability to collate these on the device is not currently a feature of GeNN. This approach allowed the implementation of a perceptron-based rule (Rosenblatt, 1958) for supervised learning described previously (Schmuker et al., 2014) and which is executed on the host machine. Note that while transferring large numbers of spikes between host and device incurs substantial demands for communication bandwidth, this hardly presents a performance bottleneck (at least not for the data volume in this application) due to the very efficient PCI-express interconnect between CPU and GPU. The learning rule is applied at the end of the presentation of each input pattern and a revised PN-AN weight matrix is uploaded to the GPU, overwriting the old one.

This cycle is repeated for the complete training dataset. Without reconstructing the model, but with the learning rule and weight update disabled, the test dataset is now presented. As before, spikes are collected per timestep. For each input the maximally spiking AN class cluster is ascertained and compared to the correct class.

### Implementation on spikey

The Spikey platform is a neuromorphic chip employing a mixed-signal approach combining analog neuron circuits with digital event routing. A detailed description of its architecture and capabilities is available in Pfeil et al. (2013). In the version that we used it supports emulation of up to 192 spiking neurons operating at a 10^4^ speedup factor, that is, 1 s biological time is executed in only 0.1 ms real time. Connectivity is unconstrained, in that any of the 192 neurons can be connected to any other. A total of 256 input driver lines are available that can serve either as spike inputs or to route spike events from internal neurons.

The Spikey PyNN-based implementation has been described in detail in Schmuker et al. (2014), including the use of inhibitory interneuron populations as per the conceptual model (Figure 1A). Important points are that, as with GeNN, the (same) plasticity rule is applied in training by workstation code acting between sample presentations. Spikes are downloaded, interrogated, and a full revised weight matrix uploaded to the device.

It is worth noting that Spikey was developed as a research platform a decade ago, and hence lacks some of the features that make current neuromorphic systems such an attractive choice for neuromorphic computing. The most drastic limitation lies in the fact that the version we used supported only 192 neurons. Hence, although the general concept of the classifier architecture is identical to the one implemented on the other platforms, compromises had to be made in the implementation. Firstly, neuron counts in all populations are much smaller; for example, we used only 6 RNs, 7 PNs, and 8 ANs per population. To achieve a robust population code with such low neuron counts we employed a Gamma process of order 5 to generate input spike trains on the workstation, which produces more regular spike trains and a lower variability in spike count than the Poisson processes used in the other implementations (see Schmuker et al., 2014 for a detailed discussion of this issue). Similarly, while the platform supports STDP (Pfeil et al., 2012), the very limited number of available spike train inputs would severely constrain, for example, the ability to generate associative learning by injecting concurrent teaching signals to the network.

### Hardware

For SpiNNaker, we used a “SpiNN-3” board that hosted 4 SpiNNaker chips with 18 ARM9 cores each. The board was connected directly to a workstation (8-core, 3.7 Ghz Intel Pentium Xeon, 32GB RAM) via 100 Mbps Ethernet. The SpiNNaker board was provided by Steve Furber's group, University of Manchester, United Kingdom. When using the connectivity profile of our classifier application, this board could accommodate up to ~10^4^ neurons and 2 × 10^7^ synapses, running in real time. The limiting factor was the number of cores available. We used the sPyNNaker software base supplied by Manchester (Rowley et al., 2015), release “Little_Rascal.”

For GeNN we used the same workstation with a nVidia Titan Black GPU card (2880 cores, 6GB memory). This is classified as a high end consumer/gaming product, connected internally via PCI-Express bus. With this card, up to ~2 × 10^4^ neurons and 4 × 10^7^ synapses running at 3 × real time could be accommodated when using the connectivity profile of our application. The limiting factor was the amount of on-card global memory available. Note that a second small video card was used to drive the workstation's main display, freeing up the main GPU. We used nVidia CUDA 7.0 and the GeNN 2.1 software release supplied by University of Sussex (Nowotny et al., 2014).

The performance measurements on Spikey were acquired via remote access to a Spikey system that was connected to a host workstation via USB in Karlheinz Meier's group at Heidelberg University, Germany. Power measurements were acquired on a second system provided by Karlheinz Meier's group that was connected to the same workstation that also hosted SpiNNaker and the GPU. The latest software version that we used was the spikey-demo package. (github.com/electronicvisions/spikey_demo, commit ea329b).

### Power measurements

For GeNN/GPU power measurement, mains electricity was supplied via a power monitor with resolution of 0.1 W to the main workstation running Ubuntu Linux with all non-essential applications closed. To obtain an average no-load baseline measurement P_BASE_, we removed the GPU card and took the power/wattage reading every 30 s for 2 min. This process was then repeated with the GPU installed to obtain a no-load “idle” power measurement for the GPU board, using the “nvidia-smi” diagnostic utility set to retrieve and log the “power.draw” parameter. During the classifier training and test, the same utility was used to log the GPU power P_GPU_ drawn every second while the power meter reading P_METER_ was used to infer the CPU power drawn during use, based on the simple formula P_CPU_ = P_METER_ − P_GPU_ − P_BASE_.

For SpiNNaker and Spikey, the external boards were also used with this same workstation, and with the GPU card removed. As before, CPU power drawn during classifier training and test was measured using the meter. To obtain measurements for the SpiNNaker hardware boards this was repeated with the board's external PSU supplied via the meter. For Spikey, a powered USB hub was employed similarly.

## Results

### Platform comparison and benchmarking strategy

Each of the classifier implementations was developed independently on each platform. It should be emphasized that our approach was to not use the smallest common feature set supported by all three platforms. Rather, we pursued the more informative approach of addressing a realistic research problem via the particular feature set of each platform. Thus, while all networks implement the same model, differences exist in their detailed operation. In consequence, parameterizations for best classifier performance differ, as presented in Table 1. The SpiNNaker and GeNN implementations were developed and tuned using only the training part of the MNIST data set and employing stratified 10-fold cross validation against all 10 digits. The test set was held unseen up to the actual benchmarking comparison. The Spikey implementation was initially developed as described previously (Schmuker et al., 2014). Due to the low neuron count, it was only trained and tested on training and test sets containing each 1000 examples of the two digits 5 and 7.

**Table 1:** **Major parameterizations of models used for each platform**.

**Parameter**	**GeNN**	**SpiNNaker**	**Spikey**
Population size (neurons)	30	30	6 (RN,PN) 8 (AN)
Presentation time per sample for learning	500 ms	120 ms	1000 ms
Inhibitory interneurons modeled	No	No	Yes
Plasticity rule	Reinforcement (off platform)	Hebbian association via STDP (on platform)	Reinforcement (off platform)

For benchmarking we explored two primary dimensions along which we varied the model parameters, the number of classes that the network is to learn and the number of virtual receptors (VRs) used to encode the data.

The number of classes (MNIST digits) requiring differentiation directly relates to the difficulty of the classification task. But increasing the number of classes also dictates that the classifier must receive and manage a correspondingly larger amount of input data, given the same number of examples per class, and accordingly needs a longer period of time for training and testing. More output spike data must also be extracted and processed for each input to determine the “winning” class (classification decision). Finally, raising the AN output cluster count increases both the neuron count and, significantly, the synapse count from the PN layer and the WTA connectivity within the AN layer.

The number of VR points assigned by the self-organizing neural gas mapping process determines the resolution of the classifier within the input space. Within the feature space, classes clustered in close proximity or overlapping will be better differentiated as the number of VRs is increased. However, this correspondingly raises both the size of the input data to the classifier and also the neuron count (rises linearly) and synapse count (rises with a square law). It has been shown previously that performance scales favorably when increasing the number of VRs, exhibiting convergence but not overfitting for large VR counts (Schmuker and Schneider, 2007). Our results confirm this observation (see below). Hence, for best classification, the network can simply be scaled up to the maximum number of VRs that are supported on the platform at hand, without running the risk of overfitting due to excessive feature counts. While scaling up the classifier does come with some cost in runtime and energy consumption, as we will see below, it is much less expensive for neuromorphic hardware than it is on classical CPU based systems. Therefore, the scaling properties of the network make it an attractive approach for massively parallel, neuromorphic systems.

### Performance in the classification task

We assessed how the model's performance scales when varying the number of digits to be discriminated and the number of VRs that are used to encode the input space. In order to provide a fair comparison for classification performance, we provided each implementation with the same training and test data, presented in the same order. Datasets for the following five combinations of digits were generated as follows; [5,7], [5,7,1], [5,7,1,9], [5,7,1,9,3], and all 10 digits [0–9]. To generate both training and test datasets, the respective MNIST dataset was traversed without shuffling or altering the order. Each allowable digit encountered was added to the dataset up to a maximum of 1000 examples of each.

For each digit combination, the number of VRs was varied between 10 and 200. The lower limit was set to the number of classes (digits). Coincidentally, 10 is also the maximum VR count that can be accommodated in Spikey without severe performance loss. The upper limit of 200 was selected because the resultant network size was the largest we were able to accommodate on the 64 core SpiNNaker “SpiNN-3” board.

**Figure 2:**
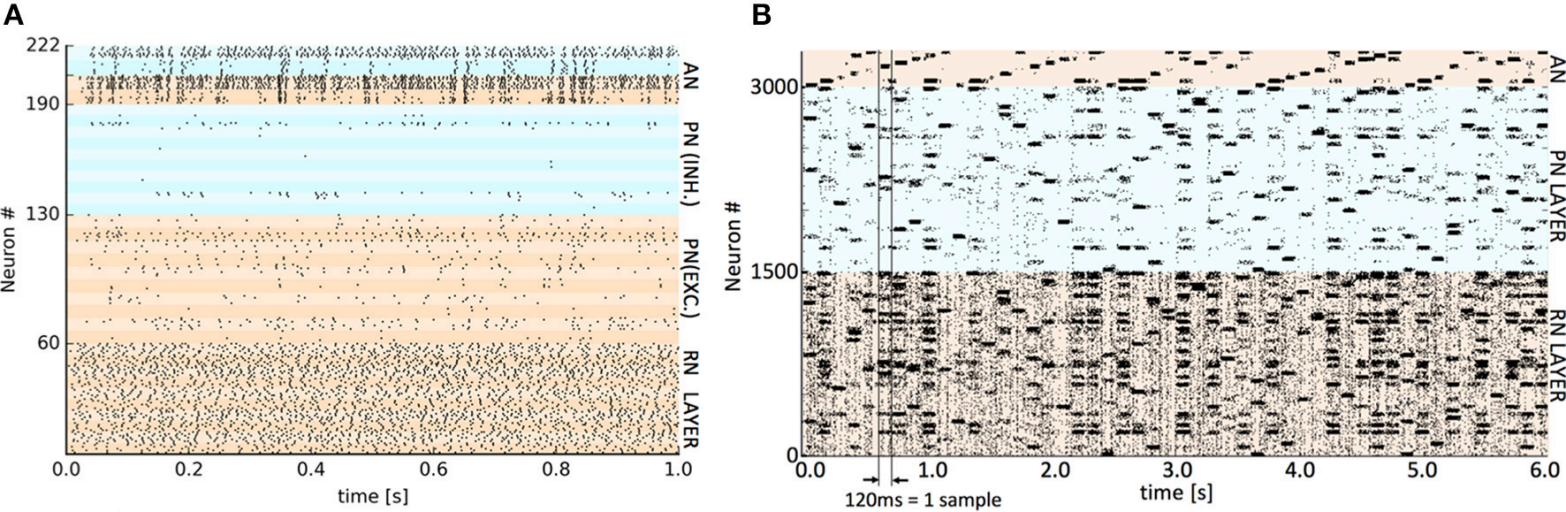
**Representative spike raster plots of classification of a test set at two levels of detail. (A)** Detail of spikes occurring during 1 s presentation of a single test digit to the trained Spikey classifier using 10 virtual receptors. The banding discriminates individual populations of 6 or 8 neurons. The colors distinguish the main layers (bottom to top): RN layer (0–59), PN and lateral inhibitory LN neurons (60–129 and 130–189), AN output neurons and paired lateral inhibitory LN neurons (190–205 and 206–222). High activity in the upper AN population determines the classification decision. **(B)** The trained SpiNNaker classifier using 50 VRs and all 10 digits (0–9). Spiking activity occurring during consecutive 120 ms presentations of 50 × MNIST test digits ordered cyclically 0–9. The colors distinguish the main layers (bottom to top): RN layer (50 clusters of 30 neurons), PN layer (50 clusters of 30 neurons) and at the top, AN output neurons (10 clusters of 30 neurons). A perfectly regular “sawtooth” pattern of activity in the output would imply 100% classification. A similar representative raster plot from GeNN is available as Supplementary Material.

The respective training datasets were used to generate the requisite set of VR points by a self-organized mapping of the input space, using the “neural gas” algorithm (Martinetz and Schulten, 1995). The same VR set was employed across the three hardware platforms to eliminate a possible source of difference. The presentation time of each sample varied by implementation (see Table 1) as both synaptic plasticity (learning) and settling time of the output activity are affected by the presentation time but this varies considerably between platforms. For SpiNNaker and GeNN this time was set empirically to the shortest period before classifying performance was adversely affected. For Spikey a nominal 1 simulated second was employed as real time spent in simulation is almost negligible on this platform. After a single run of the entire training set, plasticity was disabled (see implementation details) before presenting the test set. In all cases, the classifier's verdict was obtained by extracting a count of spikes occurring in the competing AN output populations during the presentation period of each test set sample. The winning class is assigned from the population with the highest spike count. Representative spike raster plots showing Spikey classifying a test sample digit (10 VRs, 2 classes) and SpiNNaker classifying 50 examples of the full 10 digit MNIST using 50 VRs are shown in Figure 2.

**Figure 3:**
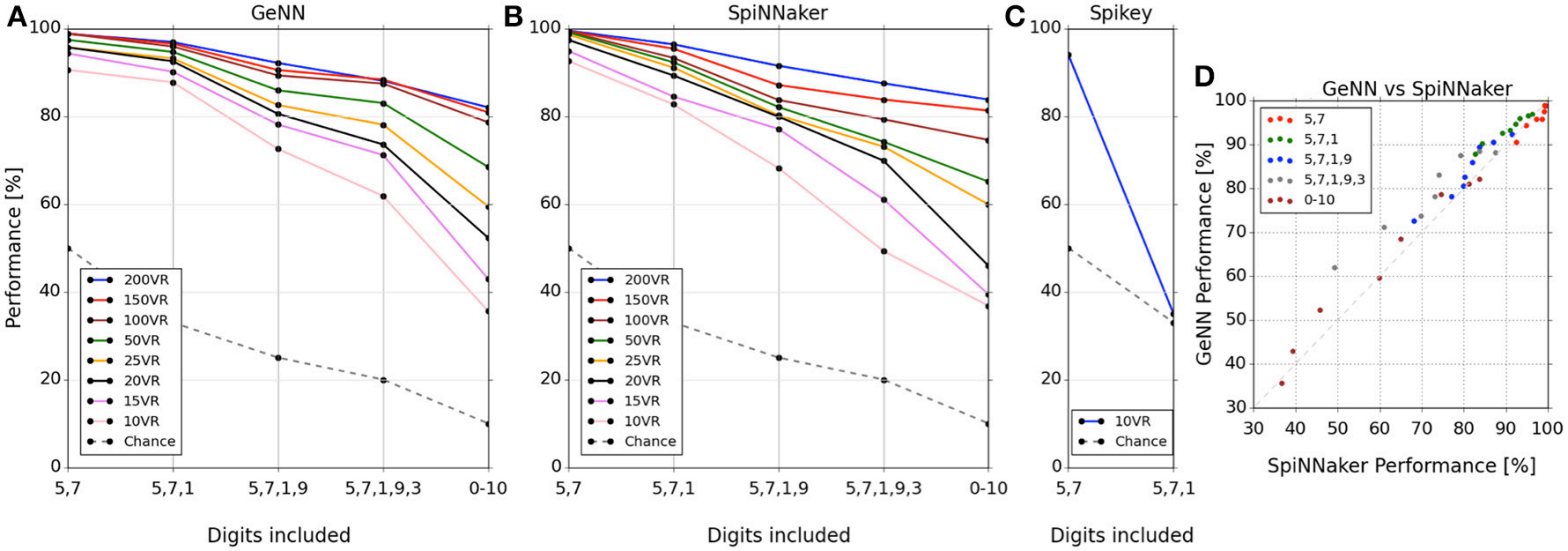
**Classifier performance on each platform for combinations of digit selections and number of virtual receptors (VRs)**. Performance is plotted as percentage correctly classified test samples **(A–C)**. **(D)** shows this performance comparatively between SpiNNaker and GeNN across all experiments. Note that the performance is similar across platforms for networks of equal size in spite of the implementation differences. Note that the lines connecting markers are included simply as a visual guide to associate results for the same number of VRs.

The classification score was measured as the percentage correctly classified from a single presentation of the test set data. The classification scores for the three platforms covering each tested combination of digits and number of VRs are plotted in Figures 3A–C respectively. Two observations can be made from these results: (1) classification gets harder as more digits are added to the task, and (2) classification performance increases as the number of VRs is increased. The latter observation points out the utility of large-scale neuromorphic systems: As more neurons become available to the algorithm, we can use more VRs to encode input space, ultimately providing a very high-dimensional representation. The massively parallel architecture of the lateral inhibition step filters out redundancy (Kasap and Schmuker, 2013; Schmuker et al., 2014) without any penalty in run time (at least on SpiNNaker or Spikey), and with a positive effect on accuracy.

The Spikey system performed slightly better than par for the smallest problem (digits 5 and 7), being tuned for this specific smaller dataset (Schmuker et al., 2014). However, performance dropped to chance levels beyond two digits as a consequence of the very low neuron count available on this system. The neuron count limits the number of VRs that can be used, as well as the population sizes. The smaller the size of a neuronal population, the lower is its ability to encode small differences in input strength via a population firing rate. This limitation is exposed by the failure of the *Spikey* network to separate three digits.

The classification performance of the GeNN and SpiNNaker implementations appear comparable, suggesting that classification performance is likely determined by the underlying classification model rather than by the details of the implementations and their optimization. To illustrate the similarity of performance in the GeNN and SpiNNaker implementations we plotted the two against each other in Figure 3D. All points are close to the diagonal indicating near-equivalent performance for GeNN and SpiNNaker implementations.

### Speed benchmarking

We next assessed the execution speed on the three platforms. We measured the total time taken for a run of the training set, then measured again for the test set. The dataset used was the same as for the classification test (see above) but restricted to the two digit {5,7}, 10 × VRs selection in order to include Spikey in the comparison. Scalability of performance was compared for the GeNN and SpiNNaker platforms by repeating the test using 100 VRs and 200 VRs.

**Figure 4:**
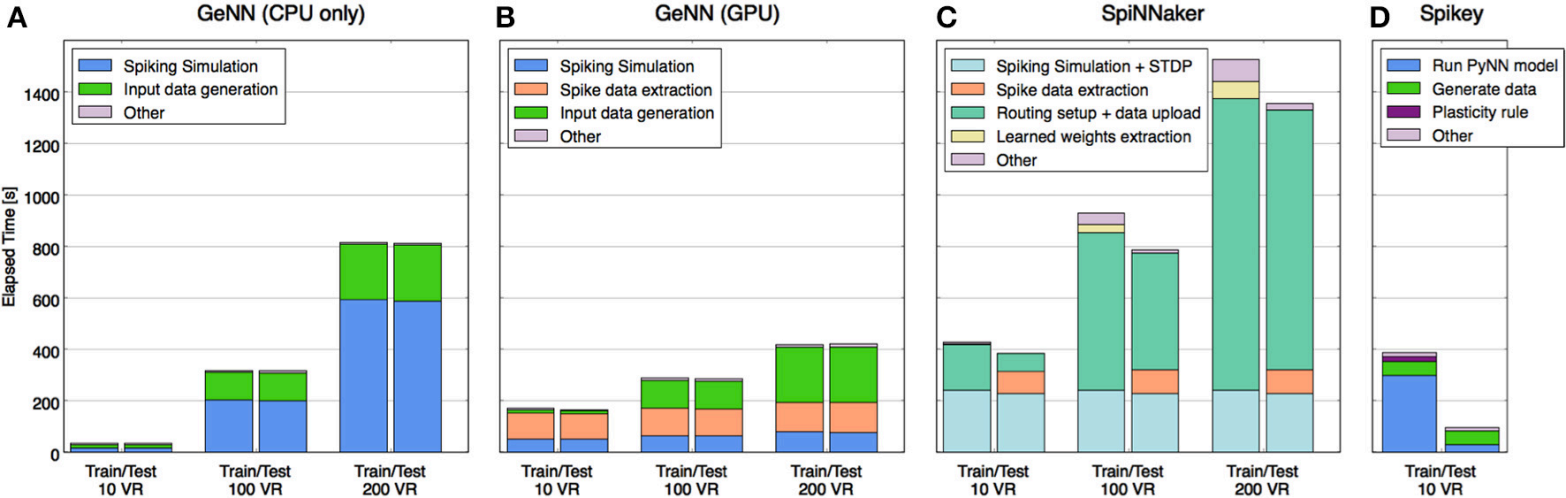
**Time taken to perform training and test phases broken down by primary tasks specific to each platform**. Tasks that required less time than could be displayed are not shown explicitly (they are grouped into “other”), e.g., the spiking simulation on Spikey. Tasks that accomplish roughly the same job on different platforms are shown in matching colors, whereas some tasks are specific to each of the platforms and are shown in distinct colors. Platforms shown: **(A)** GeNN with CPU only, **(B)** GeNN using GPU, **(C)** SpiNNaker Spinn-3, **(D)** Spikey.

In order to understand where the time is used on each platform and how this profile may change with scaling, the total time was segmented (where separate measurement was possible) into time spent on each of the following stages: model configuring/construction, input data uploading to neuromorphic platform, pure model simulation time, weight updating, spikes download/extraction. The comparative performance results and the makeup of the time expended for each platform are shown in Figure 4.

Without the requirement to communicate with a neuromorphic device, activity of the CPU-only implementation (Figure 4A) is almost entirely concerned with model simulation and generating input data (VR responses and rate-coding) for the model from the MNIST dataset. Note that simulation time increases quadratically with model size, data generation only linearly.

Figure 4B shows that the GeNN GPU implementation shifts the load of the simulation to the device but adds communication overheads, most significantly the time taken to extract spiking data from the device memory. Even with the largest model tested, this comprised a larger time component than the simulation itself, yet the time used remains almost constant with model size, suggesting that it lies well within the bandwidth limits of the interface and that the time is primarily used to marshal the call. This is unsurprising as relatively few spikes need to be retrieved every time the model is stepped.

As expected, with the simulation pegged to real time, the SpiNNaker implementation's profile (Figure 4C) is dominated at small model sizes by time spent in simulation and SpiNNaker is outperformed by the other platforms in this case. However, with the largest model of 200 VRs the ability to continue to deliver both simulation and plasticity in real time begins to show in the profile while the CPU-only implementation begins to slow down considerably. However, it is also clear that it would take a much larger model in the order of 600–800 VRs for SpiNNaker simulation time alone to outperform any of the other platforms. However, the plot also shows that supporting functionality of model setup and data uploading currently also scales with model size, raising a potential issue for this platform as a substrate for very large model simulations.

Scaling information for Spikey was not available as the number of neurons is too low to implement a larger classifier. Nevertheless, with a small model simulated at 10^4^ × real time, high performance was expected, however the end-to-end analysis (Figure 4D) shows this to be the case for the testing stage only. Studying the detailed constituents making up the PyNN run of the model (see Schmuker et al., 2014, Supplementary Material) suggests strongly that the workstation hosting the plasticity in the training stage is the root cause, specifically, the time to update the connectivity weights after every sample and the subsequent reconfiguration.

Comparing the 4 implementations for a smaller model sized for just 10 VRs, the CPU-only implementation is a clear winner over the neuromorphic platforms. This suggests three factors are at play. Firstly, the lightweight neuron models selected for GeNN and SpiNNaker do not greatly tax the CPU at low volume, compared to using relatively more computationally expensive models, such as the Hodgkin-Huxley (HH) model (Nowotny, 2011). With such lightweight models the overhead of marshaling calls to a device and managing data movements become significant factors. Secondly, a real-time simulation architecture such as SpiNNaker will also only see benefits for larger models, where other platforms cease to manage real-time or faster simulations. In our case, the CPU alone could simulate faster than real-time until the problem scale had passed 100 VRs. Thirdly, the role of a workstation controlling and interfacing with neuromorphic hardware, (for example in constructing and compiling models, preparing compatible data structures for transfer and extracting data etc.) generates a large overhead above the task of pure simulation. When a simulation model is small enough these overheads become a dominating factor.

When the model size is increased to 200 VRs a different picture begins to emerge. As expected, the serial simulation time of the CPU-only implementation increased strongly, in particular as the number of synapses increases quadratically with the number of neurons in the all-to-all PN-AN connectivity. By contrast, the parallel-executed simulation time of the other platforms has increased only little. However, we note that other workstation tasks still scale with the model and, with GeNN and particularly SpiNNaker, these now comprise an even larger component of overall time than for the smaller model. The conclusion for these platforms must be that, while the challenge of scaling the actual simulation has been addressed to a considerable extent, the scaling of associated tasks remains to be addressed better.

### Power and energy consumption

We next assessed the power consumption of the three platforms during the benchmark task. We used the two digit [5,7], 10 VR task, the same as for the speed benchmark.

**Figure 5:**
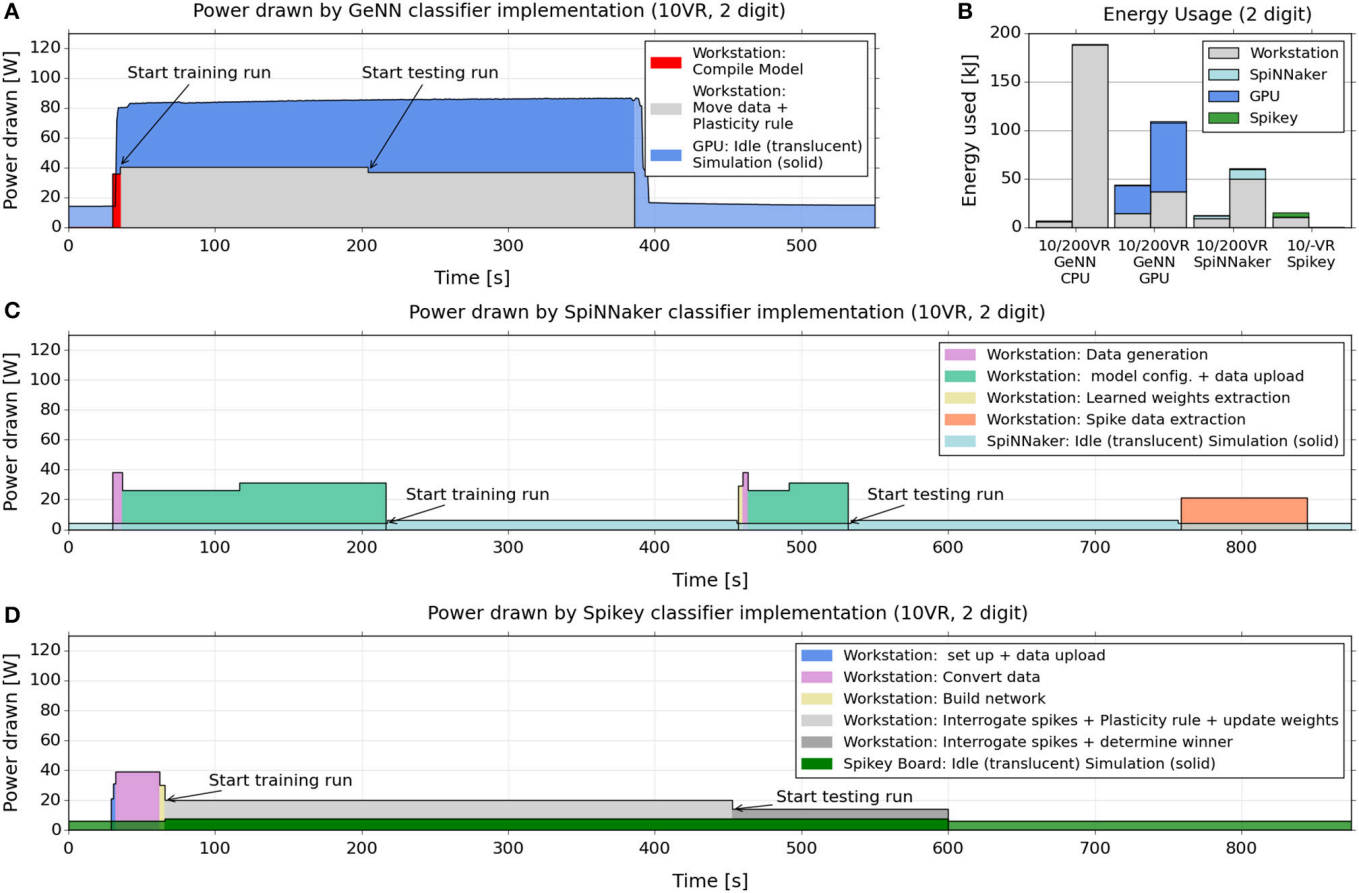
**Electrical power drawn and energy used by GeNN, SpiNNaker and Spikey across training and testing when classifying 2 digits with 10 VRs**. **(A,C,D)** power consumption across the course of training and testing of a 10 VR classifier for 2 MNIST digits (5,7), 1000 samples per digit. The graphs compare the implementations on the GeNN **(A)**, SpiNNaker **(C)**, and Spikey **(D)** platforms. We report the power drawn by the GPU card, the SpiNNaker, and Spikey boards, as well as the power drawn simultaneously by the attached workstation. The latter is reported as the power above the baseline (51 W) drawn by the workstation without GPU card and performing no task. SpiNNaker and Spikey power consumption was measured directly with the inline meter while GPU power draw was obtained via the reporting of the “nvidia-smi” utility (see main text). **(B)** Energy usage for a 10 VR and a 200 VR classifier, applied to 2 MNIST digits (5,7), 1000 samples per digit. The readings approximate the total energy (above baseline) used during the training and testing by multiplying the power usage in each stage of the process by its duration. The readings are repeated for the GeNN GPU, SpiNNaker and Spikey (10 VR only) platforms as well as for GeNN when set to use the workstation CPU alone.

Figure 5 shows how the power drawn varies over the course of the training and testing cycles, for GeNN (Figure 5A), SpiNNaker (Figure 5C), and Spikey (Figure 5D). We included the power drawn by the respective computing platforms (i.e., the SpiNNaker board or the GPU) as well as the considerable power consumed by the attached workstation during essential non-simulation associated tasks, such as uploading the model and data, and collecting and analyzing spike counts. Figure 5B indicates how the power variation with time (Figures 5A,C,D) translates to total energy expended for the classifier built using each platform. Again, each is segmented into energy consumed by the neuromorphic platform and by the attached interacting workstation.

From the plots, a similar message to the speed benchmarking emerges here, namely that a large proportion of power and energy is consumed by extended use of the workstation, in particular for what might be considered “infrastructure tasks” such as device-workstation interactions.

These interactions are not required by the CPU-only implementation, therefore energy consumption is relatively low on a small model (due to the short time required) but very high for a large model, because the simulation computation grows super-linearly with the neuron count.

For SpiNNaker, where learning or testing is first set up in full, and then runs autonomously at just 4 W without requiring the workstation, the result is a net low consumption of both power and energy, despite the longer simulation times. However, the workstation time taken to move data to and from the board results in higher CPU energy that dominates the total usage.

For Spikey, the consumption of the board is similarly very low, and the setup is relatively short but the continued involvement of the CPU for spike and weight data and plasticity rules again dominates the total energy usage.

For the GPU/GeNN implementation, the workstation is also continually involved in the plasticity rule, weight adjustment and particularly communicating with, and shuttling data to and from, the GPU device. Interestingly, however, the CPU power consumption is twice as high for GeNN than for Spikey, even though the CPU performs essentially the same task (i.e., carrying out the learning rule) in both implementations. This difference may be due to the fact that in GeNN, the CPU exchanges data with the GPU at every time-step during the simulation while on Spikey an interaction takes place only after the presentation of a digit sample, leading to less communication. Furthermore, the training phase on Spikey takes almost twice as long as on GeNN, hence the same task is spread over a longer time interval, which implies a lower power consumption if the overall energy consumption is comparable. Other differences between GeNN and Spikey that may play a role are different interconnects (PCI Express vs. USB) and different code bases (compiled code on GeNN vs. interpreted Python code for Spikey).

We note that in spite of the higher CPU power draw in GeNN, the even higher power draw of the GPU compared to Spikey or SpiNNaker hardware (some 80 W compared to 4–6 W) means that the total power and energy usage in GeNN is less dominated by the CPU as in the other cases.

## Discussion

The work described here resulted from the aim of solving a general pattern recognition task on diverse hardware and software offerings in the form they were available to us at this time. All comparisons made between the three implementations and platforms must therefore be interpreted in this context, that is complementary to isolated rote-task benchmarking statistics, that typically do not include non-essential but important details of support code and implementation specifics related to the different constraints of different platforms. We would like to suggest that studies such as the present one help to draw out and pinpoint where critical improvements may lie for a neuromorphic platform to become a strong candidate for uptake in future research projects and applications. For example, our study demonstrates that all tested platforms have great merit in accelerating spiking network emulations, but are at times let down by the overheads of code running on the attached workstation. This issue can be addressed on several levels: On the network level, emphasis should be given to minimize the communication between the device and the host machine, e.g., by exploiting on-chip mechanisms for learning as much as possible. Similarly, on the hardware side, future generations of neuromorphic systems will likely support faster interconnects that alleviate the communication bottleneck. There is likely also potential in the control software to optimize communication throughput and/or latency, and in fact this issue is currently addressed by the groups developing the interfaces to SpiNNaker and Spikey (i.e., Spikey's successor systems). Together, these measures will act to minimize the required role for the attached workstation and reduce the performance penalty for host-device communication.

### Performance of the neural classifier design as a benchmark

All three implementations delivered a similar classification performance with scores comparable to a standard neural network whilst trailing the performance of leading AI machine learning approaches such as support vector machines and deep neural networks (see http://yann.lecun.com/exdb/mnist/ for wide-ranging comparison of MNIST classification scores). As expected, performance scales with the number of VRs employed and falls with the number of classes included. The similarity of this profile, particularly illustrated between GeNN and SpiNNaker (where the majority of data was collected) suggests that the diverse implementations have succeeded in reaching the limits of the classifying ability of the underlying model. This robustness against variations in implementation and substrate suggests that the model may comprise an effective benchmark for comparing neuromorphic platforms against the other relevant criteria discussed, namely; ease of implementation, speed of processing, scalability, and power efficiency.

### Optimization targets

Our work revealed that there are some obvious targets for further development and optimization in the different platforms.

In GeNN, the high power draw of both, the CPU and particularly the GPU suggest that minimizing the time in simulation is important to reducing energy consumption. The communication overheads, in particular the total time used to extract spiking data on every timestep from the device memory, suggests that a mechanism, similar to SpiNNaker, to invoke a longer run on the device and pool spikes generated until the end would prove beneficial, at least for model sizes on the order of those tested here. The high bandwidth communication offered by the PCIe bus means that transferring large amounts of data more infrequently is considerably more efficient that transferring small amounts constantly. If spike data was collated on the card and transfers were limited to once at the end of each 500 ms (simulated time) input presentation this would still retain the ability to enact computation on the host such as bespoke plasticity rules. This argument is supported by the evidence from the time breakdown showing that the periodic upload of the updated weight data comprises only a fraction of the total time.

For SpiNNaker our results suggest that, in pure simulation performance, SpiNNaker is strong for very large neural simulations, which is what it was designed for. However, we also observed that workstation and communication functionality such as model setup and data-uploading scaled far less well. Our results make a strong case for optimization efforts that target the communication between host and device. Besides optimizations on the software side that increase throughput, also increasing the bandwidth of the Ethernet link on the SpiNNaker board may be a viable option (e.g., upgrading it to Gigabit Ethernet). It should be noted that the large SpiNNaker boards equipped with 48 x18-core chips (“SpiNN-5”) do support a high-speed AER-based interface that is available through standard SATA connectors. However, using these fast interfaces requires special hardware that is not as generally available as Ethernet.

Our work with Spikey suggests that the time to update the connectivity weights after every sample and the subsequent reconfiguration were significant performance factors in the implementation. Potentially, a large improvement in performance could result from targeting this update process in the software interface. Moreover, in future implementations one could aim at minimizing host-device communication by employing the on-chip STDP capabilities in a similar manner as in the SpiNNaker implementation, tackling the bandwidth problem at the network design level instead of just increasing the channel throughput.

### Implementation comparison—constraints or advantages from architecture, hardware, and software

The relatively much smaller capacity of the Spikey chip has clearly played a major role in guiding the model implementation on it and eventually constraining its observed classifying performance in our benchmark. The small capacity of Spikey is currently being addressed by new hardware designs (Schemmel et al., 2010). However, even though this is an obvious concern for the currently available system it is in our opinion not helpful to focus on momentary strengths and weaknesses of software and hardware aspects of the tested system in order to pick a “best” platform. Recall that the Spikey platform represents the class of accelerated hardware systems that operate several orders of magnitude faster than real-time, aiming at providing crucial speedup to long-running simulations of neuronal systems. Software interfaces can be improved and hardware designs can be modified and scaled up. Instead of entering a discussion of which approach is best overall, we would like to consider the constraints or advantages of each platform in addressing one of the main findings, that a minimized role for the workstation is potentially very advantageous. This focuses the discussion on the level of communication, data exchange and plasticity or learning required for each model implementation.

#### Data input

As GPU hardware offers high bandwidth and large memory on-device, data input for GeNN is best implemented in large chunks, avoiding the potential bottleneck, which appears when repeatedly passing small items of data across a bus. GeNN's data input functions—combined with the ability to specify a single rate code to govern spiking rates of a whole population—take advantage of this configuration, as does the ability to switch input by passing only a memory offset value. Full hardware memory state retention means that the stepwise control of the model allows new data to be uploaded whilst in simulation.

For SpiNNaker and Spikey, data input is via PyNN spike sources and needs to be stipulated per neuron for the entirety of the intended model simulation time. Addition of variable rate-coded stochastic populations would aid models of the kind attempted here. For SpiNNaker in particular, the cost of rebuilding a model means that the ability to retain state, allowing a simulation to be paused and alterations made, will prove of considerable value when it becomes generally available.

#### Running a simulation

The step-wise approach and persistent state of GeNN offers maximum flexibility to change inputs or implement additional computation or rules off-device. However, the overhead of time and power of marshaling many thousand repeated calls between the workstation and the device is considerable compared to the other platforms, which run continuously without interruption for the stipulated period. The addition of a similar “extended run” function to GeNN would be of value.

#### Implementing plasticity and learning

All three platforms offer internal spike timing plasticity. This enables simple learning at maximum speed and efficiency as the workstation is not involved, yet it proved difficult to achieve more complex learning on-platform without the introduction of somewhat convoluted teaching signal activity (SpiNNaker). Bespoke learning rules implemented off-platform require workstation time and power and introduce the overhead of transmitting updated weight data (GeNN and Spikey). Therefore, the addition, as standard, to the three platforms of extended biologically-based learning such as reinforcement learning via a form of “dopamine” reward signal and time delayed eligibility trace functionality would potentially greatly widen the opportunities for efficient simulation of learning networks. Steps in this direction are already being undertaken on the next generation of accelerated large-scale hardware systems (Friedmann et al., 2013).

#### Extracting spike data

SpiNNaker and Spikey both implement PyNN functionality to record spikes and collect them at the end of the simulation. This is an efficient approach, except where spike data is required during the run, such as for a custom learning rule run outside the platform. However, while SpiNNaker does support live spike monitoring facilities, for a custom learning rule that is implemented outside the platform, it is not enough to monitor the spikes, but one also needs to be able to updated the network by the learning rule. Implementing weight updates based on external input during the simulation would require a dedicated mechanism for interacting with the network in real time. Such a mechanism would open the door for powerful and highly flexible learning algorithms to be implemented on the hardware systems, such as three-factor learning rules that employ a reward signal driving synaptic plasticity (Izhikevich, 2007; Porr et al., 2007).

GeNN currently requires that spikes be collected on every time step, introducing a considerable overhead as discussed. We suggest that the ability to pool spike data, or spike count data, on the device would thus be of value.

### Conclusion

In conclusion, building on earlier work on each of the platforms we have demonstrated in this work that all three platforms can be used to implement a bio-inspired classifier to solve a general pattern recognition problem. All three implementations, when compared at matching size, offer comparable classification performance. Moreover, the two platforms supporting larger neuron counts exhibit very similar scaling behavior when increasing network size. This observation suggests that the functional behavior of the network was hardly affected by the considerably different routes we chose in the platform-specific implementation. On ease of use, speed and energy consumption, however, the three implementations differ considerably and in non-trivial ways. For small models a CPU-only solution appears to be best while large models are better on GeNN or SpiNNaker. However, while speed and energy consumption scale well for the actual simulation on the neuromorphic platforms, they scale much less favorably for supporting code run on the connected workstation. As neuromorphic technology matures toward general computing applications in research and technology, emphasis will have to be given to address these issues in order to bring out the neuromorphic advantage to the fullest extent.

### Conflict of interest statement

The authors declare that the research was conducted in the absence of any commercial or financial relationships that could be construed as a potential conflict of interest.
