# Supplementary material for: Comparing Neuromorphic Solutions in Action: Implementing a Bio-Inspired Solution to a Benchmark Classification Task on Three Parallel-Computing Platforms
Source: Front Neurosci. 2016 Jan 8;9:491. doi: 10.3389/fnins.2015.00491 (PMC4705229; doi:10.3389/fnins.2015.00491)
Supplement: Supplementary file 1 [file SupplementaryMaterial.PDF]

Supplementary Material

Comparing neuromorphic solutions in action: implementing a bio-inspired solution to a benchmark classification task on three parallel-computing platforms

Alan Diamond[1,*], Thomas Nowotny[1], Michael Schmuker[1]

[1] School of Engineering & Informatics, University of Sussex, Falmer, Brighton, UK

*** Correspondence:** School of Engineering & Informatics, University of Sussex, Falmer, Brighton, UK.

a.diamond@sussex.ac.uk

1 Supplementary Methods

This section adds details for the SpiNNaker implementation. Equivalent detail for the GeNN and Spikey classifiers is provided in (Schmuker et al. 2014; Diamond et al. 2015). Parameter and model settings used for the SpiNNaker are given in **Table 2**.

Table 2. SpiNNaker model parameters and settings

**Neuron + synapse models:**	Leaky integrate and fire, current based synapses ($\tau_m = 20ms, \tau_{refract} = 2ms$, $\tau_m = 20ms, \tau_{synapse_E} = 5ms,\ \tau_{synapse_I} = 5ms,\ I_{offset} = 0, V_{reset} = -70mV, V_{thresh} = -65mV$
**Weights**	Ratecode-RN: 0.4 nA, Noise-RN: 0.2 nA, RN-PN: 1.5 nA, PN-PN: 0.02 nA, PN-AN(plastic): 0.0 - 0.2 nA,<br>AN-AN: 0.06 nA, teaching signal to AN: 0.2 nA
**Connectivity**	RN-PN: 1 to 1 , PN-PN: inhibitory 40% between clusters, PN-AN: all-to-all 50%, AN-AN: inhibitory 70% between clusters, teaching signal to AN: one-to-all in correct AN cluster
**Synaptic delays**	All connections set at 1ms delay except noise-RN (set randomly 1-30ms)

1.1 SpiNNaker learning rule

PN and AN layers were connected all-to-all with random 50% connectivity with synapse weight initialized at (near) zero. During training, to create Hebbian-style associations from active VRs to a class, we used a teaching signal spike-source to trigger spiking activity in the appropriate AN

population (see **Figure 6B**). The SpiNNaker STDP implementation (see (Jin et al., 2010) for details) was enabled in the synapses afferent to the AN and the weight-change Δw curve (**Figure 6A**) was modified to obtain a mirrored positive weight gain with the same STDP window for near-coincident, pre and post-synaptic spikes of either order (pre before post or post before pre). The resultant narrow plasticity time window combined with a 20ms silence between teaching signals eliminated spillover (learning crosstalk) between input presentations.

1.2 SpiNNaker rate-coded stochastic spiking populations

Figure 6B illustrates the technique applied to create population-level rate coding in the RN layer to represent the response level of each VR by the net firing rate of a 30 neuron cluster.

To achieve this, each RN neuron is excited by 2 input sources. Firstly from a single neuron randomly selected from a larger (N=60) population providing a "noise" pool of fixed rate (100Hz) Poisson spiking neurons. Secondly, from a single spike-source-fed neuron providing a rate code. Connection weight values are set low enough to avoid RN firing from either source alone. Note that, empirically, generating a net RN firing rate of r (Hz) required firing the driving neuron at approximate 2.5 r if low afferent synapse weights are set - 0.4 (from ratecode neuron) and 0.2 (Poisson neuron). To avoid synchronization artifacts between RN neurons sharing a Poisson source neuron, we also set a random integer delay (1-30ms) on each efferent synapse leaving the Poisson population. We set a max value of r = 70Hz to match the RN firing rates in the models used by Spikey and GeNN. **Figure 6C** shows the resultant RN rate-coding (brown banding) of two consecutive 120ms input presentations to the classifier.

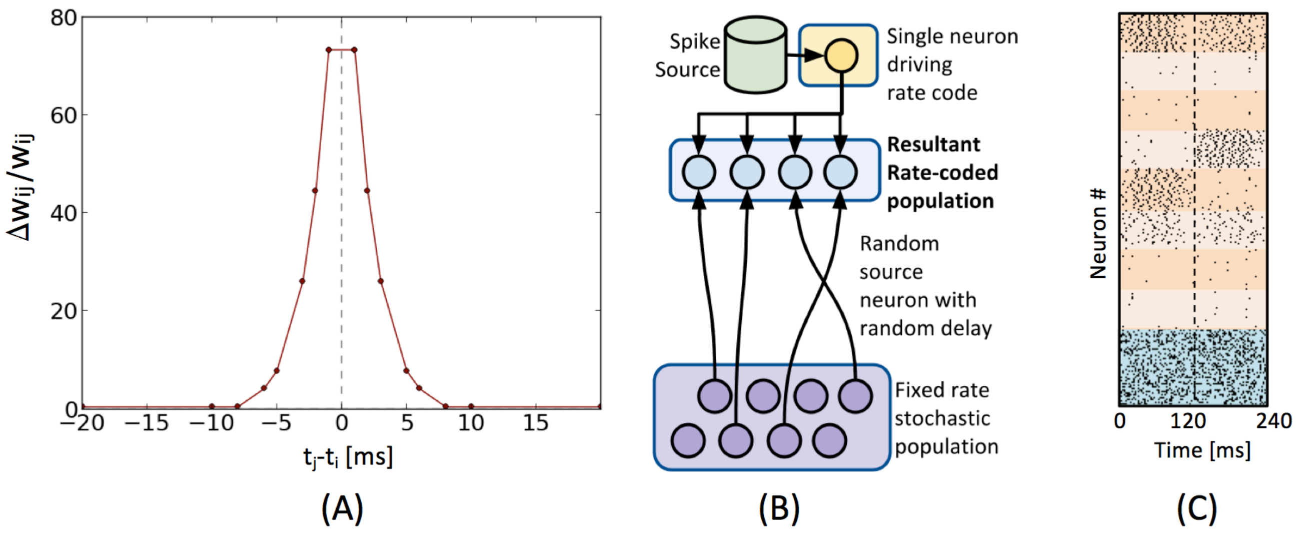

Figure 6. SpiNNaker implementation details. (A) Modified spiking timing dependent plasticity curve (after (Bi and Poo, 1998)) employed to obtain Hebbian-style learning, associating virtual receptor activity with an output class. The displayed curve shows the relative synapse weight change imposed between the ith and jth neurons, firing at times t_i and t_j respectively. (B) Approach used to creating a rate-coded population from a shared stochastic population with a fixed net firing rate and a single rate-coded neuron controlled by a PyNN spike source (see text for details). (C) Raster plot showing 2 x 120ms of activation of 10 x 30 neuron populations (brown banded zone) exhibiting net rate random spiking activity proportional to that of 10 rate coded individual neurons (not illustrated), employing sub threshold input from a subset of fixed rate Poisson firing neurons taken randomly from a 60 neuron population (blue zone) .

1.3 GeNN spiking characteristics

Figure 7 provides a representative example of the spiking response of the GeNN neuronal model to a test set example following training.

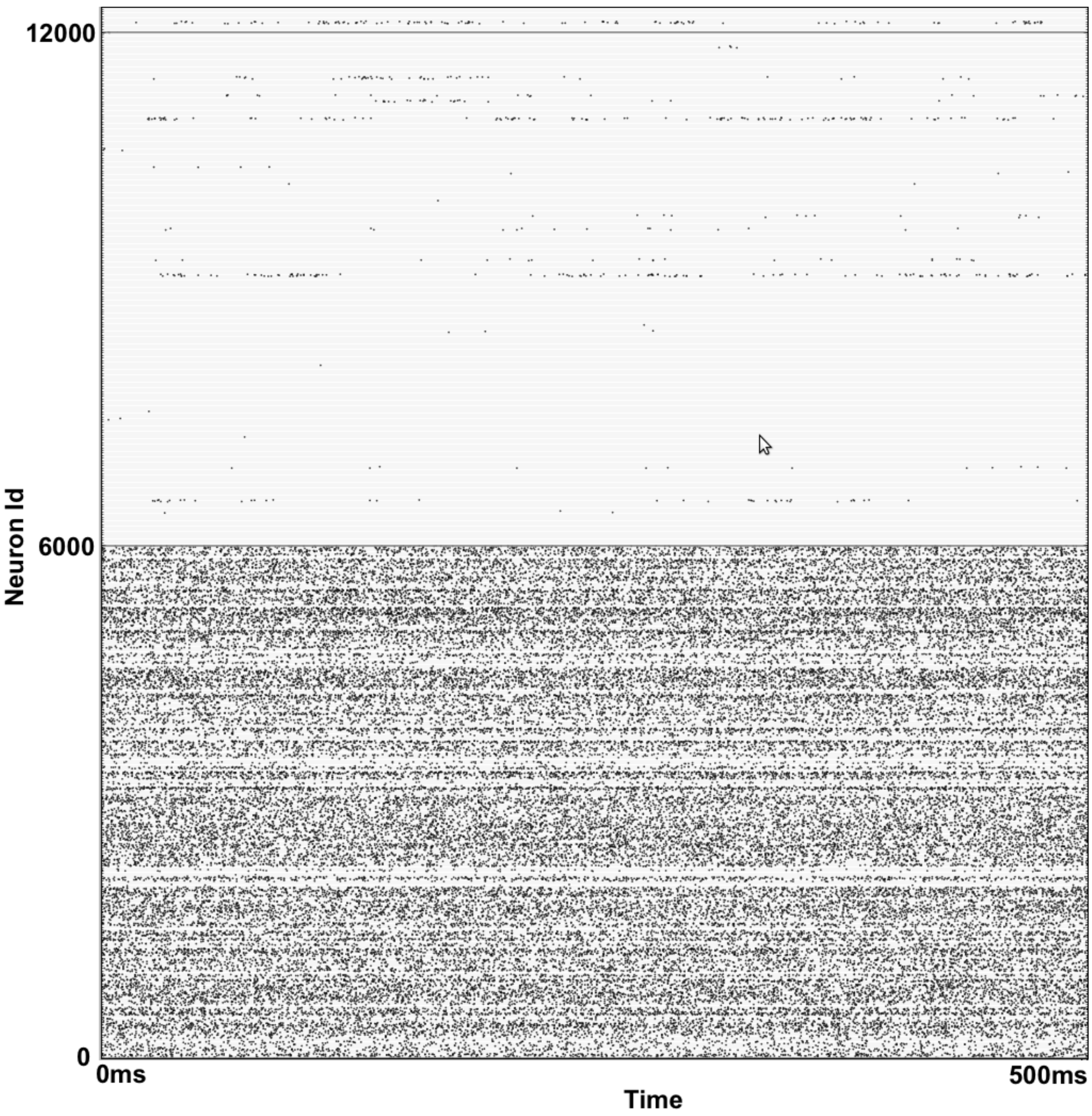

Figure 7. Representative spike raster plot of classification decision of a test set by implementation on GeNN/GPU neuromorphic hardware. The figure shows detail of spikes occurring during 0.5 second presentation of a single test digit to the Spikey classifier using 200 virtual receptors. The banding discriminates individual subpopulations of 30 neurons. The main layers (from the bottom) are RN, PN and, AN output neurons. Highest activity in a subpopulation the upper AN population determines the classification decision.

1.4 References

Bi, G. Q., and Poo, M. M. (1998). Synaptic modifications in cultured hippocampal neurons: dependence on spike timing, synaptic strength, and postsynaptic cell type. *J. Neurosci.* 18, 10464–10472.

Jin, X., Rast, A., Galluppi, F., Davies, S., and Furber, S. (2010). Implementing spike-timing-dependent plasticity on SpiNNaker neuromorphic hardware. in *The 2010 International Joint Conference on Neural Networks (IJCNN)* (IEEE), 1–8. doi:10.1109/IJCNN.2010.5596372.
